# Kidney Cancer Trends, Risk Factors, and Interventions in American Indian and Alaska Native Populations: The Kidney Cancer Association Scientific Statement

**DOI:** 10.3390/cancers18091454

**Published:** 2026-05-01

**Authors:** Salvatore La Rosa, Pavlos Msaouel, Andrew J. Sedgewick, Nathan Maulding, Alejandro Recio-Boiles, William O. Carson, Rodney C. Haring, Ken Batai

**Affiliations:** 1 Kidney Cancer Association, Houston, TX 77019, USA; slarosa@kidneycancer.org; 2 Department of Genitourinary Medical Oncology, The University of Texas MD Anderson Cancer Center, Houston, TX 77230, USA; pmsaouel@mdanderson.org; 3 Tempus AI, Chicago, IL 60654, USA; andrew.sedgewick@tempus.com (A.J.S.); nathan.maulding@tempus.com (N.M.); 4 The University of Arizona Cancer Center, Tucson, AZ 85724, USA; areciomd@arizona.edu; 5 Department of Indigenous Cancer Health, Roswell Park Comprehensive Cancer Center, Buffalo, NY 14263, USA; william.carson@roswellpark.org (W.O.C.); rodney.haring@roswellpark.org (R.C.H.); 6 Department of Cancer Prevention & Control, Roswell Park Comprehensive Cancer Center, Buffalo, NY 14263, USA

**Keywords:** renal cell carcinoma, kidney cancer epidemiology, health disparities, Indigenous People, risk factors, preventive health services, community health

## Abstract

American Indian and Alaska Native (AI/AN) populations experience disproportionately high kidney cancer incidence and mortality compared to other groups in the United States. Literature was reviewed to explore the factors contributing to the unequally higher kidney cancer burden in AI/AN communities and to develop recommendations to reduce these disparities. The incidence of kidney cancer has been rising over the past few decades, and this increase has been especially steep among AI/AN populations. Death rates in AI/AN populations are roughly twice those of the non-Hispanic White population. The elevated kidney cancer burden in AI/AN populations may be driven by both clinical and behavioral risk factors (obesity, diabetes, hypertension, chronic kidney disease, smoking, and environmental factors) and structural drivers of health, which can critically shape these disparities. Systemic inequalities limit AI/AN patients and community members’ access to chronic disease management, smoking cessation programs, primary and specialty care for early detection, and ultimately, treatment. AI/AN patients may have mistrust or other cultural barriers to engaging with the healthcare system and providers, while implicit bias in healthcare providers may lead to undertreatment. Therefore, key interventions and tailored programs aimed at reducing kidney cancer incidence and mortality are needed. Here we highlight some current interventions, including access to disease management and smoking cessation programs, facilitating healthcare access and quality, adopting patient navigation and culturally competent education, and developing strategies for early detection. In partnership with AI/AN communities, a combination of prevention, early detection, and healthcare system improvements is needed to close the kidney cancer gap.

## Simple Summary

Kidney cancer incidence and mortality rates are higher in American Indian and Alaska Native populations than in many other populations in the United States, yet the reasons for this disparity are not fully understood. This review examines factors that may contribute to these differences, including higher rates of risk factors such as obesity, diabetes, smoking, and high blood pressure, as well as challenges related to access to healthcare, early detection, and treatment. Historical, social, and economic conditions that affect health in these communities also play an important role. The goal of this work is to summarize current knowledge about kidney cancer risk and outcomes in American Indian and Alaska Native populations and to highlight opportunities for prevention, earlier diagnosis, and better care. Improving data collection, supporting community-led health programs, and increasing culturally appropriate healthcare may help reduce kidney cancer disparities and improve outcomes for these communities.

## 1. Introduction

American Indian and Alaska Native (AI/AN, or Native American) populations experience disproportionately high rates of kidney cancer incidence and mortality compared to other United States (U.S.) groups. Nationally, in 2025, the American Cancer Society (ACS) reported that kidney cancer incidence among AI/AN populations is about 90% higher than among the non-Hispanic White (here after White) population (approximately 34.2 vs. 18.0 cases per 100,000). AI/AN males have an incidence rate around 1.87 times that of White males and AI/AN females more than double that of White females [1]. This makes kidney cancer one of the most elevated cancers in this population, and it ranks as the 5th most common cancer in many AI/AN communities [2]. Correspondingly, kidney cancer death rates are considerably higher in AI/AN populations compared to White populations [2]. In Oklahoma, an area with a large Native population, two studies looking at different time periods of population data both concluded that AI/AN individuals have a kidney cancer incidence more than twice that of White individuals (32.3 vs. 15.8 per 100,000, or 2.1 times higher) and a mortality rate nearly twice as high [3,4]. These stark disparities indicate a higher burden of disease and worse outcomes in Native communities.

Several factors underlie the higher incidence of advanced-stage kidney cancer and mortality. AI/AN individuals are more likely to have lower survival, even when diagnosed early, suggesting challenges in timely diagnosis and treatment. One Arizona study found that AI/AN patients were ~30% more likely to present with advanced-stage kidney tumors, and even among early-stage cases, they had a 30% higher risk of death than White patients [5]. AI/AN populations may also be less likely to receive support when attempting to navigate the healthcare system for treating complex diseases such as kidney cancer. Multiple studies have shown the need for culturally competent patient navigation programs for AI/AN populations [6], as well as their successful implementation for cancer care [7,8]. These findings suggest that there are challenges in timely diagnosis and treatment, with the disparate outcomes pointing to gaps in healthcare access and quality that exacerbate the incidence of advanced-stage cancer diagnosis and increase mortality.

The objective of this narrative review is to explore the factors contributing to an unequally higher kidney cancer burden in the AI/AN communities in the U.S. We conducted PubMed and Google searches to identify any recent (preferably 2010 or later) academic papers, government documents, and web resources from academic institutions and community organizations related to kidney cancer in AI/AN communities and other specific topics discussed in this paper. We also reviewed papers citing key papers on kidney cancer in AI/AN populations. Our literature review suggests that the increased kidney cancer burden in AI/AN is driven by a combination of greater exposure to risk factors and systemic inequalities. The information presented in this review may guide interventions to reduce kidney cancer burden in these communities.

## 2. Characterizing the AI/AN Population and Limitations in Surveillance Data

### 2.1. AI/AN Population Characteristics

The AI/AN population, when utilizing the U.S. Census definition, comprises a heterogeneous group representing more than 570 federally recognized Tribal Nations (often described as tribes, villages, pueblos, and nations) across the U.S., as well as more than 100 state recognized Tribal Nations, unrecognized Tribes, and numerous Indigenous Peoples from across the Americas. Each group has distinct cultural, linguistic, and historical identities. For this review, we will be using the term AI/AN throughout, as the majority of research in this field relies on U.S. Census data, and we will seek to use the same terminology to ensure the most appropriate statistics. Currently, the U.S. Census does not have tools to delineate U.S.-based Indigenous populations from those hailing from other parts of the Americas in their AI/AN category. AI/AN is not merely a racial category as used in the U.S. Census. Tribal Nations are sovereign entities within the U.S., and many have and operate their own government and healthcare system. However, there are individuals who identify themselves as AI/AN or reported as AI/AN in the Census without affiliation with tribal Nations or communities.

According to the 2020 U.S. Census, approximately 9.7 million individuals identified as AI/AN alone or in combination with another race. This population is younger on average than the White population (median age: 31 vs. 41 years) and is more likely to reside in rural or medically underserved areas, including tribal lands and reservations. These structural and geographic factors contribute to persistent disparities in healthcare access and outcomes. AI/AN communities bear a disproportionate burden of chronic conditions, including cancer, driven by multiple factors, such as poverty, environmental exposures, limited access to specialty care, and chronic underfunding of the Indian Health Service (IHS) [9,10].

### 2.2. Limitations of Data Related to Cancer Incidence and Mortality in AI/AN Populations

Despite these documented disparities, the interpretation of cancer statistics in AI/AN populations is limited by several methodological challenges. A major issue is the persistent racial misclassification of AI/AN individuals in cancer registries, leading to an underestimation of cancer incidence and mortality rates [11]. A recent systematic review on racial misclassification with AI/AN populations noted that 55 of 66 studies included direct issues related to misclassification, and this issue is commonly addressed through linkage with IHS and tribal health clinic records [12]. This issue may partly stem from how AI/AN groups are recognized as both a racial category and political status for members of Tribal Nations [13], thus creating confusion in the general public over who constitutes being and/or appearing like an AI/AN person. Additionally, most available statistics rely on extrapolations from datasets that either incorporate Indian Health Service linkage or are restricted to Purchased/Referred Care Delivery Areas (PRCDAs), which may not fully represent urban or non-IHS-affiliated AI/AN populations [10,14,15].

## 3. Trends in Kidney Cancer Incidence and Mortality

### 3.1. Incidence Trends

**Figure 1:**
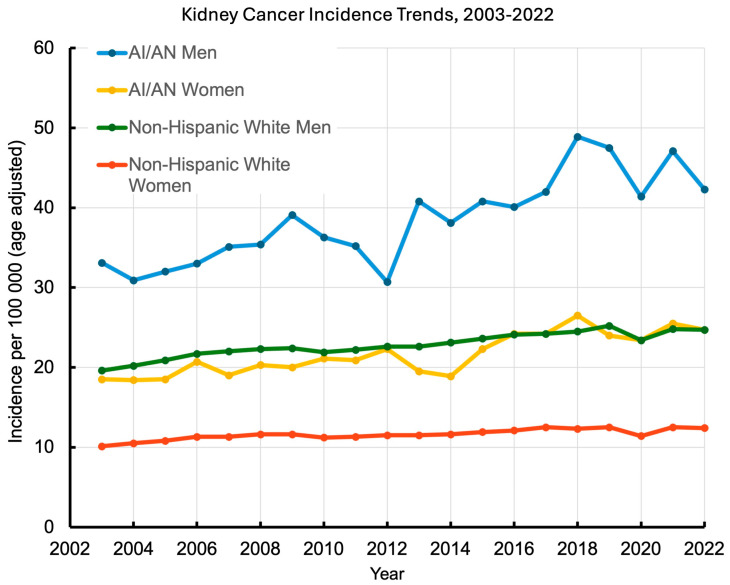
Trends in age-adjusted kidney cancer incidence among American Indian/Alaska Native (AI/AN) and non-Hispanic White adults, United States, between 1999 and 2020. Lines display annual age-adjusted incidence rates per 100,000 population for AI/AN men (blue), AI/AN women (orange), White men (green), and White women (vermillion). Rates derived from North American Association of Central Cancer Registries (NAACCR) Cancer in North America (CiNA) Explorer [19]. Over the study period, incidence increased fastest in AI/AN men, widening the disparity relative to Whites, while AI/AN women showed a steeper rise than White women.

Kidney cancer incidence has been rising over the past few decades in the U.S., and this increase has been especially steep among AI/AN individuals (Figure 1). Data indicate that since the late 1990s, kidney cancer rates in AI/AN populations have climbed rapidly. From 2009 to 2018 alone, the incidence in AI/AN populations rose approximately 2.8% per year, more than double the annual increase seen in White populations (+1.1% per year) [16]. Similarly, a national analysis from 1999–2019 found kidney cancer incidence to be rising much faster in both AI/AN men (+2.7% per year) and women (+2.4% per year) than White men (+1.7 per year) and women (+1.7 per year) [10]. This widening gap suggests that disparities have grown. Kidney cancer rates in AI/AN populations are not only higher to begin with but also increasing faster than in White populations. The trend is evident in various regions. For instance, the cancer registry in California showed no decline in kidney cancer incidence among AI/AN individuals from 2000 to 2016, even as some cancers decreased among White individuals [17]. In fact, kidney cancer was one of the few cancers for which the incidence increased significantly each year among California AI/AN individuals (approximately +4.7% annually), compared to a smaller +1.8% yearly increase among White individuals [17]. These trends coincide with the increasing prevalence of obesity and diabetes in many AI/AN communities over that period, suggesting a link between these factors. Another factor in the rising incidence is improved race-specific data accuracy. Earlier undercounting of AI/AN individuals in cancer registries has been corrected through linkage with IHS records, revealing higher true rates [16]. The age-specific pattern is particularly concerning: analyses of national data from 1999–2019 found that the largest average annual percent increases in NH AI/AN kidney cancer incidence occurred among individuals under age 50 for both sexes (+5.2 per year in AI/AN vs. +2.9 per year in NHW males and +3.7 per year in AI/AN and +2.9 per year in NHW females), and AI/AN patients are disproportionately represented among early-onset RCC cases diagnosed before age 50 in the National Cancer Database and in regional registries [10,18].

### 3.2. Mortality Trends

On its own, the increase in kidney cancer incidence among AI/AN populations could simply reflect earlier detection resulting in more diagnoses without a corresponding rise in mortality. However, mortality (death rates) from kidney cancer reflects both incidence and survival and has remained elevated in AI/AN populations. National cancer statistics (2014–2018) show that kidney cancer death rates in AI/AN populations are roughly twice those of Whites [2]. Historically, some improvements in kidney cancer survival have occurred due to better treatments and earlier detection in the general population. However, AI/AN individuals have not benefited equally from these gains. While specific long-term trend data for AI/AN kidney cancer mortality are limited, partly due to past racial misclassification issues, analyses of kidney cancer mortality rates have consistently indicated that the mortality disparity has persisted [20]. Nationally, the five-year survival for kidney cancer between 2011 and 2017 is lower in AI/AN than in Whites (71% vs. 76%) [1]. While the kidney cancer mortality rate is declining in both the AI/AN and White populations, the decline is slower in the AI/AN than White population (−0.6 vs. −1.9 annually) [2]. In Oklahoma, from 1997 to 2017, kidney cancer mortality among Natives was nearly two times higher than in Whites [3]. In Arizona, AI/AN kidney cancer patients diagnosed between 2007 and 2016 have a 33% increased risk of overall mortality compared to White patients, even after adjusting for neighborhood-level socioeconomic factors [5]. This gap may largely be attributed to later diagnosis and treatment inequalities.

## 4. Association with Specific Kidney Cancer Histological Subtypes

Most renal cell carcinomas (RCCs) are of the clear cell subtype, but roughly one-quarter present with variant histologies (papillary, chromophobe, renal medullary carcinoma [RMC], other molecularly defined subtypes, or unclassified tumors) [21]. Each subtype harbors distinct metabolic hallmarks and oncogenic drivers associated with specific risk factors [22,23]. Clear cell RCC, for instance, depends heavily on imported cholesterol to sustain PI3K/AKT signaling and control reactive oxygen species [24]. RMC is driven by *SMARCB1* loss, which imparts resistance to hypoxic stress [25], whereas chromophobe RCC exhibits mitochondrial alterations that disrupt oxidative phosphorylation, heightening its reliance on the tricarboxylic-acid cycle [26].

Clear cell RCC is an obesity-associated subtype and is the dominant histology profile in AI/AN patients [27,28,29]. Analyses of the National Cancer Database and Arizona Cancer Registry data found that 86.3% and 88.7% of AI/AN kidney cancer patients have clear cell RCC [18]. A single-institution study that included a small number of AI/AN patients reported that approximately 90% of tumors in AI/AN patients were clear cell and about 83% in the same cohort overall [30,31]. The second and third most common RCC histologies overall, papillary and chromophobe RCC, have not been frequently described to date among AI/AN patients but likely together account for most of the remainder RCC histologies in this population. More rare subtypes, such as RMC, are extraordinarily rare because sickle hemoglobinopathies, such as the sickle cell trait, a major risk factor for RMC, are virtually absent in most AI/AN populations [32,33].

## 5. Key Risk Factors Driving Higher Kidney Cancer Rates

Multiple risk factors, both modifiable and non-modifiable risk factors, are thought to contribute to the higher kidney cancer incidence observed in AI/AN populations. However, no studies to date have specifically assessed how these risk factors affect kidney cancer incidence within AI/AN communities. Much of the current understanding is extrapolated from research conducted in other populations in the U.S. or internationally.

### 5.1. Modifiable Risk Factors

**Figure 2:**
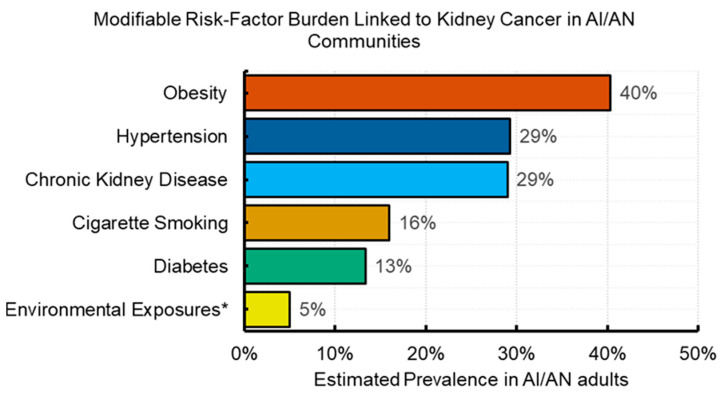
Prevalence of major modifiable risk factors for kidney cancer in American Indian/Alaska Native (AI/AN) adults. Data are drawn from recent national surveillance sources—National Health Interview Survey (NHIS) [34] for hypertension, smoking, diabetes, and obesity; Kidney Early Evaluation Program (KEEP) [35] for Chronic Kidney Disease; and studies for environmental contaminants. The asterisk that “Environmental Exposures” are hotspot-specific rather than uniformly distributed across all AI/AN adults, and that the 5% value aggregates several distinct contaminants.

Modifiable risk factors include comorbid conditions that are consequence of lifestyle factors, lifestyle and environmental factors (Figure 2, Table 1). Below is a breakdown of major modifiable risk factors, ranked by their estimated impact in the general population (mainly in White population), along with supporting data.

#### 5.1.1. Excess Body Weight (Obesity)

Obesity is one of the most significant contributors to kidney cancer, predominantly the clear cell RCC subtype, and obesity in early- and mid-life increases the risk of developing kidney cancer later in life [27,36,51]. It alters hormones and growth factors in the body, promoting RCC. Excess weight is estimated to account for roughly 30–35% of kidney cancer cases in the general population [9,36,37,38]. AI/AN adults have some of the highest obesity rates in the nation—in many communities, >40% of adults are obese, significantly higher than the U.S. average [39]. This elevated prevalence of obesity may be a major driver of the higher kidney cancer incidence in AI/AN populations. In fact, the pattern of higher AI/AN kidney cancer rates “likely reflects the higher prevalence” of obesity in this group [9]. Other cancers with well-established connection with obesity reflect this trend, even if their incidence in AI/AN vs. White individuals varies [52] with liver [53] having the highest (2.5×) rate ratio, followed by gallbladder [54] (2.4×), kidney [36] (1.9×), colon and rectum [55] (1.4×), esophagus [56] (1.1×), corpus and uterus [57] (1.1×), and pancreas [58] (1.05×).

#### 5.1.2. Commercial Tobacco Use (Smoking)

Although all forms of tobacco, including traditional or natural tobacco used for ceremonials can produce carcinogens when burned, there is a substantial difference between traditional and commercial tobacco. This starts with the pattern of use of traditional tobacco that tends to be occasional, not habitual, and often involves small quantities, to the fact that commercial tobacco products instead contain hundreds of additives, many of which are additional carcinogens and toxins that are not present in natural ceremonial tobacco. Health organizations (including the Centers for Disease Control and Prevention [CDC] and National Native Network) emphasize the important distinction between traditional and commercial tobacco and advise against conflating cultural use with recreational smoking risks [59,60]. Smoking commercial tobacco is a well-established risk factor for kidney cancer, causing DNA damage in kidney cells. Long-term smokers have a substantially higher risk of RCC (risk increases with duration and intensity of smoking). About 16–20% of kidney cancers may be attributable to smoking in recent estimates [37,40]. AI/AN communities have the highest smoking rates of any U.S. racial and ethnic group, which amplifies their kidney cancer risk. Recent data show that 16–35% of AI/AN adults smoke cigarettes, while the rates for White adults are much lower (11–13%) [41]. AI/AN smokers also have barriers to accessing smoking cessation resources and have lower quit rates [61]. This high prevalence of commercial tobacco product use is likely a key factor in the elevated kidney cancer burden. Vaping or e-cigarette is sometimes used as a replacement of commercial tobacco as smokers attempt to quit smoking, but vaping is becoming common among youth in AI/AN communities as in other communities [62,63,64]. However, the long-term effect of vaping on cancer, particularly kidney cancer is still unknown.

#### 5.1.3. High Blood Pressure (Hypertension)

Hypertension is an independent risk factor for kidney cancer. Elevated blood pressure may directly affect kidney tissue or correlate with other metabolic risks. People with hypertension have a higher likelihood of developing RCC even if on medication [65,66,67,68]. About 27% of kidney cancers may be attributable to hypertension [42,43]. Hypertension is common in AI/AN adults, partly linked to high rates of obesity and diabetes. Some studies have noted a higher prevalence of hypertension in AI/AN populations than in White populations, which likely contributes to their increased kidney cancer risk [44].

#### 5.1.4. Diabetes

Diabetes and related insulin resistance may promote carcinogenesis in the kidney, independent of obesity [66,69]. Diabetes increases the risk of RCC by 40–70% in some prospective cohort studies, even after accounting for body weight [45,46,47]. AI/AN populations have the highest type 2 diabetes rates in the U.S., with a prevalence 50% or even two-fold higher than in White populations [48,70,71]. This is significant because diabetes elevates kidney cancer risk even beyond the effect of obesity. Poorly controlled diabetes can also lead to chronic kidney disease (CKD), further compounding the cancer risk. The high diabetes burden in many AI/AN communities may thus be a major contributor to kidney cancer incidence, although further research is needed to elucidate this relationship.

#### 5.1.5. Chronic Kidney Disease and Dialysis

Individuals with advanced kidney disease or on long-term dialysis have an increased risk of RCC due to chronic renal stress and acquired cystic changes [43,72]. Patients on dialysis for many years have a risk of kidney cancer several-fold higher than the general population [49,73]. CKD accounts for 8–10% of kidney cases [42,49]. AI/AN populations suffer high rates of CKD and end-stage renal disease (ESRD), largely due to diabetes and hypertension. For instance, in 2021, the incidence of ESRD among AI/AN individuals was 2.3 times that of White individuals, the prevalence was over twice as high as among White individuals, they had the lowest percentage with a kidney transplant (19.4%), and they the highest percentage receiving in-center hemodialysis (71.1%) [50]. This means that more AI/AN patients require dialysis, putting them at risk for kidney tumors. Improved management of diabetes and blood pressure to prevent kidney failure would also reduce RCC cases.

#### 5.1.6. Environmental Exposures (Water and Industrial Toxins)

Certain environmental contaminants prevalent in some regions increase the kidney cancer risk [66,74,75]. Notably, inorganic arsenic in drinking water is a carcinogen linked to kidney and other cancers. Even low-level arsenic exposure (below the U.S. Environmental Protection Agency [EPA]’s 10 ppb limit) is associated with a higher RCC risk. One study found a 22% increase in kidney cancer risk in areas with >5 ppb arsenic in water and a 4% risk increase for each doubling of the arsenic concentration [76]. Many AI/AN communities rely on well water or local water systems that historically had arsenic or other contaminants. For example, parts of the Northern Plains and Southwest with high concentrations of AI/AN residents have naturally high arsenic and uranium in groundwater, affecting tribal communities [77,78,79]. AI/AN participants in the Strong Heart Study living in these regions had high levels of arsenic, uranium, and other heavy metals in their urine [80]. Additionally, legacy uranium mining on Navajo Nation and other lands has led to uranium and radon exposure, and industrial pollutants (like cadmium or trichloroethylene from certain workplaces) can also play a role [65,81]. These environmental risks are region-specific but significant where present. Ongoing efforts to ensure safe drinking water in AI/AN communities aim to reduce this risk factor [82,83]. Industrial exposures may cluster regionally, too. As another example, in Alaska and the Plains, some Native people work in mining, oil, or agriculture jobs with exposure to solvents (like trichloroethylene) or heavy metals that are linked to RCC [84]. Such exposures can contribute to local cancer disparities not seen elsewhere.

### 5.2. Non-Modifiable Risk Factors

Established non-modifiable risk factors of kidney cancer are AI/AN individuals, genetics and familial factors, sex, and age. Currently, there is no evidence suggesting that non-modifiable risk factors are unevenly affecting AI/AN individuals.

#### 5.2.1. Genetic and Familial Factors

A family history of kidney cancer or certain hereditary syndromes (such as von Hippel–Lindau, hereditary papillary RCC, Birt–Hogg-Dubé) greatly increases risk [85,86,87]. These genetic conditions are rare but confer a very high lifetime RCC risk. Having a first-degree relative with kidney cancer can also modestly raise one’s risk [65,66,69]. There is no evidence of a unique genetic predisposition to kidney cancer in AI/AN populations broadly. While hereditary RCC syndromes can cluster in any family regardless of background, no specific familial or tribal clustering of these syndromes has been documented in AI/AN populations to date [88]. Overall, genetic factors likely account for a small fraction of kidney cancers in AI/AN populations. Hereditary kidney cancer due to pathogenic genetic variations in RCC-related genes, such as *VHL*, accounts only for a small proportion of RCC cases (3–8%) [89,90]. These genetic variants are rare in many populations (frequency of <1%). Although it is necessary to investigate frequencies of these rare pathogenetic variants in AI/AN patients to reach a conclusion, it is unlikely that these rare variants exist in such a high frequency in AI/AN populations. Common genetic variants of kidney cancer susceptibility genes also account only for a small proportion of kidney cancer cases. From common genetic variants identified in a multi-ancestry genome-wide association study meta-analysis [91], the heritability of kidney cancer was calculated to be 16.4% in European-ancestry populations. The genetic risk score calculated using these genetic variants only slightly improved risk prediction, with the area under the curve (AUC) from 0.71 to 0.74 for kidney cancer and from 0.70 to 0.74 for clear cell RCC, from baseline information (sex, age, body mass index, smoking, hypertension, and background genomic variation). Frequencies of genetic variants that increase kidney cancer risk are not higher in 1000 Genome Project Indigenous American populations. However, it remains important to recognize and surveil high-risk families.

#### 5.2.2. Sex and Age

While not unique to A/AN individuals, it is important to note that kidney cancer is about twice as common in men as in women, and the risk increases with age as most cases occur after age 50 [66,69]. AI/AN men, therefore, are expected to experience higher incidence rates than AI/AN women, due to this general male predisposition and historically higher smoking rates among men. Additionally, the AI/AN populations have a younger median age compared to White populations (early 30 s vs. 40 s) [9]. Since kidney cancer risk rises with age, this younger age structure would be expected to result in a lower incidence. The fact that AI/AN communities still have higher kidney cancer rates despite being younger overall highlights the impact of other risk factors on kidney cancer incidence. Notably, the incidence of kidney cancer in men is much more rapidly increasing than in women (Figure 1). The reasons for the steeper male trajectory are unlikely to reflect crude obesity prevalence per se, because the prevalence of BMI-defined obesity in AI/AN adults is not much higher in men than in women [92]. The steeper male trajectory, therefore, may reflect a convergence of sex-differential factors such as fat distribution, smoking, hypertension, and occupational and environmental exposures layered on the already ~2:1 baseline male predominance of RCC.

Although dedicated studies in AI/AN communities are scarce, data suggest that the higher burden of kidney cancer in AI/AN populations is largely driven by modifiable lifestyle factors, which have been impacted by the constant underfunding of Tribal Nation governments and IHS [93]. These factors include examples such as obesity, smoking, and comorbid conditions like diabetes and hypertension, all of which are more common in this population [9]. Physical inactivity is also linked to these modifiable lifestyle risk factors, but a potentially independently increased kidney cancer risk [94,95]. Environmental exposures in certain regions further exacerbate the risk. While genetic factors are also expected to contribute, the interplay of lifestyle and social factors creates a perfect storm leading to elevated kidney cancer rates in AI/AN communities.

It should be noted that while this section focuses on risk factors for developing kidney cancer, some of these same factors also worsen prognosis by elevating the risks of recurrence and mortality. The link between obesity and oncologic outcomes remains to be fully elucidated, while evidence suggesting smoking, hypertension, and diabetes increasing the risk of mortality is accumulating [96,97,98]. Closing this gap in kidney cancer burden will require tackling major reversible risk factors, such as obesity and tobacco use, but must also address gaps in funding and healthcare access, as well as underlying factors that increase the prevalence of modifiable risk factors. Expanding the funding and access to Indigenous patient navigators for cancer services has evidence of effective intervention and should be considered for further efforts [99,100].

## 6. Structural and Social Determinants (Or Drivers) of Health and Their Role in Disparities

Structural and social determinants (or drivers) of health (SSDOH), the societal conditions that people are born to and live in that influence many aspects of health and wellbeing, are underlying risk factors that critically shape kidney cancer disparities in AI/AN communities. The structural systems, upstream factors such as political, legal, cultural, and economic systems, create inequalities in social conditions, which drive health disparities [101]. Centuries of systemic inequalities and racism have created a social environment where many AI/AN individuals face socioeconomic and healthcare challenges that indirectly boost cancer risk and worsen outcomes [102]. Key SSDOH and social contextual factors are discussed below.

### 6.1. Poverty and Education

AI/AN communities have disproportionately high poverty rates. More than one-fourth of the AI/AN population lives in poverty, and especially, AI/AN women report the highest rate of about 1 in 4 women living in poverty [103,104]. Lower income and education levels limit access to healthy food, stable housing, and health literacy, which in turn foster the lifestyle risks (poor diet, smoking, and physical inactivity) linked to kidney cancer. Economic hardship and low health literacy can also delay people from seeking medical care until illnesses are advanced.

### 6.2. Healthcare Access and Insurance

Access to high-quality and comprehensive healthcare is a major determinant of cancer outcomes. AI/AN populations often lack adequate access due to geographic isolation and underfunding of the IHS, which serves many AI/AN communities. A recent U.S. Census Bureau report mentioned that 18.8% of AI/AN individuals are uninsured, far above the 5.7% rate among White individuals [105]. Even those with IHS access may find local clinics understaffed or lacking specialty care. A significant challenge in the implementation of healthcare for Tribal Nations is that a sizeable portion of funding is appropriated annually, with no assurances of funding after that. Each new administration comes to office with differing political goals, and the funding is susceptible to changes. The IHS has been chronically underfunded for FY 2025, Congress approved a budget of $8.2 billion for the Indian Health Service, an increase of $1.1 billion or 16% above FY 2023. Of the total, roughly $1 billion was set aside for the referred-care program and $260 million in proposed mandatory funding for the special diabetes program for Indians [106]. On the other end, a committee of tribal health and government leaders has long made funding recommendations that far exceed the agency’s budget. The latest report from the National Indian Health Board (NIHB) states that the IHS needs $63 billion to cover patients’ needs for the fiscal year 2026, including $10 billion for referred care [107]. The most recent federal budget for 2026 did increase funding for the IHS, but only an increase of 1.3%, far short of the amount needed to properly support the agency [108]. Despite all of the cuts to federal programs that support AI/AN health initiatives outside of the IHS, the Trump administration put forth a proposed 2027 budget, which again increases the IHS budget, this time by over 1 billion dollars. This is a needed but still confusing set of political policies [108].

As a result, preventive services like cancer screening and routine primary care are thus less available. In fact, only about 56% of AI/AN adults are up-to-date on colorectal cancer screening (as a proxy for preventive care engagement), versus 69% of Whites [9]. Without specialty care at the IHS facilities, many AI/AN patients are referred to non-IHS facilities for imaging or other assessments, adding to an already lengthy diagnostic process. This gap in preventative and specialty care suggests that AI/AN patients may have fewer opportunities for incidental early detection (detecting an asymptomatic kidney tumor on an imaging scan done for another reason). Surgical and oncology care for RCC is also referred to non-IHS facilities making cancer care coordination difficult. Limited access and the availability of preventive care also mean risk factors, such as hypertension and diabetes, may be undertreated, compounding the cancer risk. Additionally, AI/AN individuals are over twice as likely to be uninsured as Whites individuals (27% vs. 10%) [9]. Taken together, these SSDOH factors increase exposure to lifestyle-related risks (e.g., limited access to tobacco prevention, diabetes, and weight management programs), delay diagnosis, and limit access to effective treatment in a timely manner. Ultimately, these drive both higher incidence and mortality in AI/AN populations.

### 6.3. Rural Isolation

A significant portion of the AI/AN population lives in rural areas or on reservations. There is shortage of urologists and oncologists across the U.S. [109,110], and this shortage is more pronounced in rural areas [111,112]. Surgical care for urologic cancer is also becoming centralized, and more surgical care, particularly minimally invasive surgery, is performed in high-volume medical centers usually located in urban and metropolitan areas [113,114]. On the other hand, over half (54%) of AI/AN people reside in rural or small-town settings, where healthcare infrastructure is sparse [115]. Specialist care (e.g., urologists or oncologists) and advanced imaging facilities may be hours away. This geographic barrier adds to the financial burden with additional costs for travel, hotel stays, and missing work, leading to delays in diagnosis and treatment. For instance, a person with hematuria (a warning sign of kidney cancer) in a remote community might postpone getting a proper workup due to travel and logistics. By the time they present, the cancer could be more advanced. Moreover, clinical trials are less available in rural communities, and AI/AN populations have low clinical trial enrollment rates, partly due to structural barriers related to living rural areas [116,117]. Rural health disparities also intersect with technology as fewer local prevention programs or health awareness campaigns reach these areas.

### 6.4. Historical Mistrust and Cultural Barriers

Past abuses in research and healthcare have fostered the mistrust of medical institutions among some AI/AN individuals [118,119]. Cultural differences and a lack of AI/AN representation among healthcare providers can result in communication gaps. These factors may cause some AI/AN patients to avoid or delay seeking care or to decline recommended interventions. Additionally, language or cultural nuances (especially for elders or those in very traditional communities) might impede an understanding of health messages, unless delivered in a culturally tailored way. All these can contribute to later-stage diagnoses, delayed treatment initiation, forgoing treatment altogether, and low clinical trial enrollment.

### 6.5. Bias and Discrimination

Systemic bias has impacted the social and economic opportunities for AI/AN individuals, and interpersonal bias can affect the care they receive. Implicit bias and discrimination in healthcare providers is common and may lead to poor quality of care (undertreatment or different treatment decisions and recommendations) [120,121,122]. For example, clinicians may dismiss an AI/AN patient’s symptoms or withhold aggressive cancer treatments because of preconceptions about adherence to follow-up care [123]. Such disparities in care quality directly influence survival. The lower kidney cancer survival in AI/AN individuals, even when they are diagnosed at a similar stage as Whites, suggests possible differences in treatment or comorbidity management. Studies found that AI/AN patients with Stage I RCC were less likely to receive surgical treatment [124,125]. Another study noted that even when kidney tumors are caught early, AI/AN patients had worse survival, hinting at unequal access to optimal surgical or oncologic treatment [5].

The SSDOH in AI/AN communities creates the context in which multiple factors, such as high poverty, limited healthy food options (leading to obesity), high smoking rates partly due to targeted tobacco advertising [126] and stress, inadequate healthcare access, and long distances to providers, all feed into a higher kidney cancer risk. These same factors lead to later detection and treatment disparities, raising mortality. Addressing kidney cancer in AI/AN populations therefore requires not just a medical approach but also social and structural solutions to improve living conditions and healthcare equity [127].

A note on the international versus within-country patterning of kidney cancer is warranted here. At the ecological level, kidney cancer incidence is higher in high-income than in low- and middle-income countries [128], a pattern largely attributable to a greater obesity prevalence, longer life expectancy, cumulative tobacco exposure, and widespread cross-sectional imaging leading to the incidental detection of small renal masses [66,69,129]. Within high-income countries such as the U.S., however, this relationship inverts at the individual and community level. Kidney cancer incidence, an advanced stage at diagnosis, and mortality are consistently higher in individuals with a low socioeconomic status and from a high neighborhood disadvantage, reflecting the clustering of established RCC risk factors—obesity, commercial tobacco use, hypertension, diabetes, CKD, and environmental exposures—in socioeconomically disadvantaged populations, compounded by delayed presentation and reduced access to guideline-concordant care [5,31,37,125,130,131]. AI/AN communities have the highest poverty rates of any U.S. racial and ethnic group (with approximately one in four AI/AN individuals living below the federal poverty level) [103,104], the lowest median household incomes, and the most limited healthcare access, and these conditions co-localize geographically with the highest regional kidney cancer incidence [10]. Although dedicated analyses of kidney cancer incidence stratified by income within AI/AN populations are not yet available, the existing ecological and regional evidence is consistent with a gradient in which the most economically disadvantaged AI/AN communities bear the heaviest kidney cancer burden.

## 7. Geographic and Regional Disparities

Kidney cancer disparities in AI/AN populations are not uniform across all areas—they vary considerably by geographic region, influenced by differences in tribal demographics, local environments, and healthcare infrastructure. Some regions and counties with high AI/AN populations have especially elevated incidence and worse outcomes.

### 7.1. Regional Variations (IHS Regions)

The IHS divides the country into regions, and cancer data show wide regional swings in kidney cancer rates among AI/AN (Table 2). In general, the Northern Plains and Southern Plains regions have the highest kidney cancer incidence in AI/AN, whereas the Southwest and East have lower rates. For example, incidence rates among AI/AN males range from about 19 per 100,000 in the East to ~65 per 100,000 in the Northern Plains—a more than three-fold difference. AI/AN women show a smaller regional range (around 19.5 in the Southwest up to ~27 per 100,000 in Northern Plains) [10]. These patterns mirror regional disparities in risk factors and socioeconomic conditions. The Plains regions (e.g., the Dakotas, Nebraska, and Oklahoma) have historically high smoking rates and obesity prevalences, which likely drive their higher kidney cancer burden. In contrast, the Southwestern region (e.g., Arizona and New Mexico), despite challenges like arsenic exposure, has had lower overall cancer rates possibly due to lower tobacco use historically [132]. It is notable that in almost all IHS regions, AI/AN men and women have higher kidney cancer incidence than Whites. The East is the main exception, where White rates are relatively high and AI/AN rates are comparatively low [10]. This suggests that the regional environment and lifestyle affect all races, but AI/AN communities start at a disadvantage in most areas.

### 7.2. County-Level Hotspots

Certain counties with large AI/AN populations show pronounced disparities. San Bernardino County (California) is one example often cited. It is home to a significant urban AI/AN community and several tribes. In California statewide data, AI/AN females had a 56% higher kidney cancer incidence than White females (19.1 vs. 12.3 per 100,000) [17], and Southern California counties like San Bernardino contribute to this gap. Factors in San Bernardino and similar counties include pockets of poverty, environmental exposures, and potentially less access to specialized care for AI/AN residents. Despite being in an urban area, AI/AN individuals might face barriers within the larger healthcare system. Other county hotspots include those overlapping large reservations or tribal lands, for instance, Apache and Navajo Counties in Arizona, which cover much of the Navajo Nation; McKinley County, New Mexico (Navajo and Zuni); and counties in Oklahoma with high AI/AN populations (such as Cherokee County). These areas often report elevated kidney cancer incidence or mortality among AI/AN, reflecting local risk factor concentrations [4,10,133]. In Oklahoma, the state cancer registry confirms significantly higher kidney cancer rates for AI/AN vs. Whites statewide—aligning with the fact that Oklahoma (Southern Plains) has among the highest incidence for AI/AN nationally [3].

### 7.3. Healthcare Access for Urban Populations

AI/AN individuals living on reservations (e.g., in parts of Alaska, the Southwest, or the Northern Plains) may have severely limited healthcare facilities, as described in the previous section. Despite closer proximity to healthcare facilities, AI/AN individuals living in urban areas, such as Los Angeles, Phoenix, and Tulsa, may still face access barriers (like lack of insurance as well as structural and cultural/language barriers) [134,135]. These differences can affect outcomes. For instance, previous research observed that urban AI/AN populations have elevated kidney cancer incidence rates compared with urban White populations [10]. Although there is no specific study for kidney cancer, another study in an urban healthcare setting in California Bay area with similar access to preventive care and treatment showed that urban AI/AN individuals had worse survival for some cancers compared with urban White individuals [136]. San Bernardino County and other urban counties (e.g., Los Angeles and Maricopa in AZ) illustrate this dichotomy. They have larger absolute numbers of AI/AN patients and major cancer centers available, yet disparities persist, implying that socioeconomic and cultural barriers within urban settings remain a challenge.

In summary, where AI/AN individuals live strongly influences their kidney cancer risk and outcomes. Regions like the Northern/Southern Plains and certain high-AI/AN population counties bear a particularly heavy burden, due to a confluence of high risk-factor prevalence and weaker healthcare resources. Meanwhile, regions like the East (with smaller, more dispersed AI/AN populations and generally better access to care) see smaller disparities. Recognizing these geographic patterns allows public health efforts to target “hotspot” areas—for example, focusing interventions in counties with high AI/AN populations (San Bernardino, Apache, McKinley, various Oklahoma counties, etc.) and tailoring strategies to local needs (such as addressing arsenic in water on the Plains, or improving care coordination in urban Indian health clinics).

## 8. Interventions to Reduce Kidney Cancer Incidence and Mortality

Addressing the higher kidney cancer incidence and mortality in AI/AN communities requires a comprehensive, multi-level approach. Key interventions span the prevention of risk factors, early detection, and improvements in healthcare delivery. Below, we discuss evidence-based or promising interventions, along with their rationale, effectiveness, and feasibility in AI/AN contexts (Table 3). While we strongly recommend expanding these interventions to reduce risk factors and improve healthcare access and kidney cancer care, effects of these interventions on reducing kidney cancer incidence and mortality in AI/AN populations and gaps in the kidney cancer burden are still unknown.

### 8.1. Tobacco Control and Cessation Programs

Reducing commercial tobacco use is arguably the single most impactful intervention for preventing many cancers, including kidney cancer. Programs that provide culturally tailored smoking cessation support for AI/AN smokers have shown success in improving quit rates. For example, community-driven campaigns that distinguish sacred/traditional tobacco use from commercial smoking, combined with counseling and nicotine-replacement therapy, can resonate well [60].

Effectiveness: Smoking cessation yields substantial health benefits—the risk of RCC gradually declines after quitting (though it may take over a decade for risk to significantly drop) [155]. Given the very high smoking prevalence in AI/AN (where up to 1 in 3 adults smoke in some regions), even modest reductions can prevent numerous cancers.

Feasibility: Challenges include addressing addiction in communities where tobacco use is socially normative and coping related. However, feasibility is bolstered by existing public health frameworks (IHS offers cessation programs, and the CDC’s Tips From Former Smokers campaign has specific AI/AN testimonials). Prioritizing tobacco cessation through increased funding for tribal tobacco control programs, enforcing smoke-free policies on tribal lands, and engaging youth with anti-tobacco education is a high-priority, cost-effective strategy to lower kidney (and other) cancer rates.

Recent Efforts: The Great Plains Tribal Cancer Control Program (2023–2027) identified kidney cancer among the top five cancers in the Northen Plains tribe’s region. Thirty-five percent of current adults in the Great Plains area are smokers. The plan aims at highlighting and raising awareness about important cancer issues, challenges, and barriers faced by this population with the ultimate goal of reducing the incidence and mortality rates of cancer in these communities by working collaboratively with the tribes, organizations, and local entities [137]. At Roswell Park Comprehensive Cancer Center, the Department of Indigenous Cancer Health adapted Tobacco Treatment Specialist Programs for Indigenous communities, including all seven within the state of New York [142,156]. Other efforts, like the Cherokee Nation Cancer Registry [138,139] and the Improving Cancer Outcomes in Native American Communities (ICON) initiative in Oklahoma [140,141], are gathering local data and implementing interventions (e.g., tobacco cessation, patient navigation, tele-oncology) to address not only kidney, but also other high-burden cancers.

### 8.2. Obesity- and Diabetes-Prevention Initiatives

Given the role of obesity and diabetes in kidney cancer, lifestyle interventions are critical. Research with Tribal Nations has shown that initiatives targeting diabetes prevention based on AI/AN culture can reduce risk for diabetes. The National Diabetes Prevention Program (DPP), federally funded through Nation to Nation agreements have been shown to decrease and prevent diabetes [157,158]. The IHS funded by Congress implemented the Special Diabetes Program for Indians Diabetes Prevention (SDPI-DP) involving over 300 communities across 35 states and 12 IHS administrative. Proven programs like the DPP, when adapted for AI/AN communities (as done by the IHS SDPI-DP), have led to weight loss and reduced progression to diabetes [159]. These programs promote healthy eating (including returning to more traditional diets high in fiber and lean game and low in processed foods) and regular physical activity.

Effectiveness: Lifestyle interventions can reduce body weight by 5–7% and drastically cut diabetes incidence—this, in turn, is likely to reduce obesity-related cancers.

Feasibility: Such programs require sustained funding and community buy-in. Feasibility is improved by involving tribal health departments, using community health workers, and leveraging cultural strengths (e.g., traditional dancing as exercise, community gardens for healthy food). Many AI/AN communities have shown enthusiasm for wellness programs when they are community-led. Addressing food insecurity (e.g., by subsidizing fruits/vegetables and improving grocery access on reservations) is also an important complement to make healthy eating feasible [160].

Recent efforts: after implementation of the SDPI-DP in 1997, between 1999 and 2013, the incidence rate of ESRD due to diabetes in AI/AN people fell by 54%, a greater decline than for any other racial or ethnic group. Additionally, from 2013 to 2017, diabetes prevalence among AI/AN adults decreased consistently, indicating a reduction in new cases [143].

We must also touch on the new market for weight-loss-assistance drugs, glucagon-like peptide-1 receptor agonists (GLP1s), for aiding in the management of type 2 diabetes and obesity. These are shown to be effective but also costly. A recent study documented that without insurance, and with manufacturer discounts, a monthly supply of GLP1 may cost an individual between $373 and $717 [161]. This would make it almost impossible for AI/AN individuals with limited income or no insurance to afford the medication, despite the need given the high rates of obesity and type 2 diabetes within the community. The cultural appropriateness and acceptability of weight loss drugs also need to be evaluated.

### 8.3. Hypertension and Kidney Disease Management

Strengthening routine primary care and chronic disease management in Native health systems will help control hypertension, treat diabetes, and monitor kidney health—thereby reducing long-term cancer risk. Interventions include blood pressure screening at every clinic visit, the aggressive management of high blood pressure (with culturally acceptable patient education about medications and diet), and early treatment of kidney disease. For patients with CKD or on dialysis, regular ultrasound screening of the kidneys can allow the early detection of RCC (some nephrology units perform annual ultrasounds after 3–5 years on dialysis, as those patients are high-risk) [162].

Effectiveness: Good blood pressure control and the use of ACE-inhibitor medications can protect the kidneys and may lower RCC risk indirectly.

Feasibility: The feasibility hinges on improving healthcare access (discussed below) and ensuring that IHS and tribal clinics have resources for chronic care follow-up. Task-shifting strategies, such as training community health workers to do home blood pressure checks and telemedicine consults with nephrologists, have been piloted in some rural AI/AN communities with positive outcomes.

Recent efforts: The Target: BP™ initiative, launched by the American Heart Association and American Medical Association in 2017, supports Federally Qualified Health Centers—many serving AI/AN communities—with no-cost tools, training, and recognition to improve hypertension control. Nearly half of the participating sites are these centers, facing challenges similar to the IHS and tribally operated facilities. As part of the National Hypertension Control Initiative, a three-year Health Resources and Services Administration (HRSA) and Office of Minority Health grant, the American Heart Association trained and assisted 350 HRSA-funded health centers with pre-pandemic BP control rates below 58.9%. From 2017–2022, over 3100 organizations participated, serving 8 million patients; 42% achieved control ≥70%. These strategies are directly applicable to AI/AN settings, helping prevent CKD progression and lower the RCC risk [144].

### 8.4. Environmental Health Interventions

In areas where environmental exposures elevate risk, targeted interventions can substantially reduce harm. Increasing awareness, identifying exposure-sources, community monitoring and flagging exposure, and rapid mitigation to reduce exposure require a multi-layer commitment from an engaged community, tribal leadership, and state and federal support. A prime example is providing arsenic-free drinking water. In the Northern Plains, a recent community-led project installed under-sink arsenic filtration systems in homes on reservations with contaminated wells [163]. This intervention, paired with education, has been effective in lowering arsenic levels in the water consumed by families. Nevertheless, the long-term and scalable impact will require coordinated policy reforms to address contamination more broadly.

Effectiveness: Removing arsenic exposure should reduce the long-term risk of kidney cancer and other diseases—one study suggests that even low arsenic levels raise kidney cancer risk by 6–22%, so eliminating that exposure could correspondingly reduce the risk [76]. Similarly, ongoing efforts to remediate uranium mine waste on Navajo Nation land and provide safe water sources are expected to improve kidney health outcomes.

Feasibility: Infrastructure projects like water filtration or connecting homes to safe water systems require investment but are highly feasible with government and tribal support, and many are underway supported by the U.S. EPA and National Institutes of Health (NIH) grants. Another environmental intervention is regulating workplace exposures: ensuring that AI/AN individuals working in industries like mining have proper protection from solvents/heavy metals (via the Occupational Safety and Health Administration [OSHA] enforcement and tribal advocacy) can prevent occupational RCC cases. These interventions often need collaboration among tribes, federal agencies, and researchers but have shown success when the community is involved in planning (as with the Strong Heart Study and Strong Heart Water Study’s partnerships) [82,145].

Recent efforts: In February 2021, the EPA issued the “Federal Actions to Address Uranium Contamination on Navajo Nation 2020–2029,” also known as the Ten-Year Plan. The plan includes the cleanup of abandoned uranium mines, disposing of mine waste and monitoring of groundwater contaminants, access to safe drinking water, and training of health care providers on the health effects of non-occupational exposure to uranium and on documentation of exposure history [81].

### 8.5. Improving Healthcare Access and Quality

Perhaps the most pivotal intervention is to strengthen healthcare delivery for AI/AN communities to catch cancers earlier and treat them optimally. This involves multiple components:

#### 8.5.1. Increase Funding and Capacity of IHS/Tribal Health Facilities

Adequately fund the IHS to at least meet basic healthcare needs—this would expand the clinic hours, staffing, and availability of diagnostic services. More funding can also support bringing specialists (urologists and oncologists) to remote areas via regular outreach clinics or telehealth. Congress’s recent moves to increase IHS budgets on FY 2025 are steps in the right direction [164], but the recent setback from the current administration to freeze or cut the IHS budget could disrupt Urban Indian Organizations not to be able to sustain operations and critical services as early as within 30 days or less without federal funding [165].

Effectiveness: Better-funded facilities can provide timely imaging (e.g., ultrasound or CT scans when patients have symptoms like flank pain or hematuria), leading to earlier kidney cancer diagnoses. They can also facilitate prompt referrals for surgery. For example, if a small renal tumor is found, having the resources to get the patient to a nephrectomy or ablation quickly can be lifesaving.

#### 8.5.2. Expand Insurance Coverage

Encouraging Medicaid expansion in states with large AI/AN populations (many of which, like South Dakota and Oklahoma, historically had not expanded Medicaid until recently) will lower the uninsured rate. With insurance, AI/AN patients can access a broader network of providers beyond the IHS. Evidence shows that insurance coverage improves cancer survival by enabling earlier treatment.

Feasibility: Policy changes are involved, but tribes and public health groups have been lobbying successfully for Medicaid expansion and enhanced Medicare coverage for AI/AN elders.

Recent efforts: Tribal leaders have engaged in government-to-government consultations with state Medicaid agencies to co-design policies that respect tribal sovereignty and address the unique healthcare needs of AI/AN populations. These collaborations have led to innovative Medicaid programs in states like Washington and Arizona, aiming to improve health outcomes for AI/AN communities [146,147].

#### 8.5.3. Patient Navigation and Community Health Representatives

Implement patient navigator programs to guide AI/AN cancer patients through the healthcare system. Trained navigators (often Native themselves and/or familiar with the culture/language) can help patients schedule appointments, overcome logistical hurdles (transportation and lodging), and understand their treatment options [166]. Such programs have proven effective in other cancer disparities—navigators have increased adherence to treatment and follow-up in Native communities for cancers like breast and colorectal [167]. For kidney cancer, a navigator could ensure, for example, that a patient diagnosed at an IHS facility gets connected to a high-quality surgical and cancer center and returns for follow-ups.

Feasibility: Many cancer centers (including National Cancer Institute-designated centers) now partner with AI/AN communities to provide navigation services, and some grant funding (NIH or ACS) is available for this. The key is building trust—hiring from within the community and respecting traditional healing practices alongside medical treatment.

Recent efforts: A notable example is the University of Oklahoma’s Stephenson Cancer Center, which received a $17.2 million NIH grant to collaborate with 16 tribal nations. The project focuses on cancer prevention, screening, and care coordination, with an emphasis on culturally tailored navigation services. The center’s AI/AN navigators have already served over 4460 AI/AN patients, representing 65 tribal affiliations [148]. Roswell Park Comprehensive Cancer Center has also developed an Indigenous & Rural Patient Navigation Program that provides free, non-clinical services to assist patients from cancer screenings through survivorship. The program employs trained community members as navigators to offer culturally competent support, including connections to screening services, information about cancer topics, and referrals to necessary services [149]. The navigation program at Roswell Park Comprehensive Cancer Center has been funded by the Bristol Meyers Squibb Foundation, and this illustrates the importance of diversifying founding sources for successful long-term patient navigation programs.

#### 8.5.4. Culturally Competent Education and Outreach

Increasing awareness about kidney cancer in AI/AN communities can promote the earlier evaluation of symptoms. Outreach might include educational materials in Native languages about warning signs (blood in urine, persistent back pain) and risk factors. Also, public health messaging that emphasizes reclaiming healthy traditional lifestyles (e.g., “return to ancestral foods to fight modern diseases”) can indirectly support cancer prevention. Some tribes host annual health fairs or use local radio to spread such messages.

Effectiveness: Education alone is not a panacea, but it can shift norms over time. For instance, if more people understand that smoking and obesity are linked to cancers, community-led norms may slowly change, as seen in some tribes that have passed tobacco-free workplace laws and organized weight loss challenges. These changes have been observed for how Indigenous People globally, including AI/AN, Maori, First Nations in Canada, and Native Hawaiians, have successfully implemented employee assistance programming built on having a sense of community to support in challenging times [168].

Recent efforts: Many organizations, tribal groups, and local government agencies are conducting culturally competent education and outreach initiatives that even if not directly targeted to kidney cancer, address some of its root causes. Many have already been mentioned; other notable efforts include the Fred Hutchinson Cancer Center’s CANOE Partnership: The Cancer Awareness, Navigation, Outreach, and Equitable Indigenous Health Outcomes (CANOE) program focuses on smoking cessation and lung cancer screening [150]. The Messengers for Health: Based on the Crow Reservation in Montana, this organization, led by Alma Knows His Gun McCormick [151], provides community-based health education that integrates traditional Crow beliefs and practices, enhancing the effectiveness of cancer prevention messages.

### 8.6. Early Detection Strategies

Unlike breast or cervical cancer, there is no standard screening test for kidney cancer in asymptomatic people. However, early detection can still be improved via incidental findings and targeted approaches:

#### 8.6.1. Incidental Detection

Many kidney tumors are found “incidentally”, often at an earlier stage on imaging (CT or ultrasound) done for other reasons. Ensuring that AI/AN patients have access to such imaging when medically appropriate is important. For example, if an AI/AN patient is in a high-risk category (middle-aged man with a long smoking history and obesity) and presents with abdominal pain, advocating for an ultrasound or CT could catch a tumor that might otherwise go unnoticed. Some clinicians in high-disparity areas are being more vigilant in imaging high-risk AI/AN patients. Additionally, lung cancer screening with low-dose CT is now recommended for heavy smokers; these scans can sometimes pick up kidney lesions [169,170]. Enrolling all eligible AI/AN smokers in lung screening programs might yield the collateral benefit of kidney tumor detection.

#### 8.6.2. Targeted “Screening” Initiatives

Although no country currently operates a dedicated national CT or ultrasound-based screening program for kidney cancer, given the very high incidence in certain AI/AN subgroups, researchers have suggested exploring pilot screening programs. For instance, the idea of ultrasound screening in high-incidence regions (like parts of Oklahoma or the Northern Plains) has been floated [4,171]. This would involve scanning at-risk individuals (say, AI/AN men over 50 with multiple risk factors) to detect small, asymptomatic kidney cancers.

Effectiveness: It is unproven for the general population because RCC is less common than other screened cancers, but in a targeted high-risk group, it might be cost-effective and worth further investigation. Any formal screening proposal in young AI/AN adults would need to be preceded by prospective feasibility and cost-effectiveness studies that explicitly address the cumulative radiation dose, overdiagnosis of indolent small renal masses, community acceptability, and equitable downstream access to urologic and oncologic care—without which a screening program could paradoxically widen rather than narrow disparities.

Feasibility: This would need careful planning and resources, as well as community acceptance (ultrasound is non-invasive and generally acceptable). While not the standard of care yet, it is an area for research and could be trialed through IHS if supported by data. Data in support could be obtained by the Cherokee Nation Cancer Registry, the first Surveillance, Epidemiology, and End Results (SEER)-affiliated tribal registry that provides localized, population-specific data on kidney cancer incidence guiding the identification of high-risk individuals and geographic hotspots [138,139]. In the meantime, strengthening existing screening programs for other cancers (colon, cervical, lung) in Native communities is crucial—not only do those save lives directly, but having robust screening infrastructure builds trust and health system engagement that can spill over to the better management of all conditions, including kidney cancer.

#### 8.6.3. Genetic Testing

An emerging area for cancer prevention and screening programming is biomarker and genetic testing to better assist in early detection for hard-to-detect cancers [172]. Genetics research may be potentially difficult to do with Indigenous Populations due to past transgressions and violations of Indigenous Peoples Rights such as with Arizona State researchers secretly stealing Havasupai genetics data for decades of research [173]. Current research is built upon community engagement, research coordination, consent, policy around data protections and storage of biospecimens, and the willingness to learn [172].

### 8.7. Interventions in High-Risk Communities

In locales like San Bernardino County or the Navajo Nation, where local issues drive risk, tailored interventions are needed.

#### 8.7.1. San Bernardino (Southern California Urban)

The San Manuel Indian Health Clinic, part of Riverside–San Bernardino County Indian Health, Inc. (RSBCIHI, Grand Terrace, CA, U.S.), provides culturally sensitive healthcare services, including medical, dental, and behavioral health care. Collaborations between RSBCIHI and local healthcare institutions aim to bridge gaps in specialty care access for Native populations in the region. To combat food insecurity and promote nutrition, RSBCIHI offers food and nutrition programs, including the Commodities Food Distribution program, which provides food packages with frozen meats and fresh produce to eligible AI/AN households [152].

#### 8.7.2. Northern Plain Reservations

Environmental health interventions (arsenic filtration, monitoring of well water) can improve safe-water use reducing exposures to the environmental toxin that increase kidney cancer risk [82,145]. By enhancing tele-oncology services, patients diagnosed with cancer can consult experts without traveling far [154]. Also, initiatives to curb the extremely high smoking rates among Northern Plains tribes (which are among the highest in the country) are vital [153]; this could include stricter policies on tobacco sales on reservations and youth prevention programs, given the widening gap in smoking prevalence between AI/AN and White individuals.

#### 8.7.3. Oklahoma and Southern Plains

Since data show very high incidence and mortality [3], a comprehensive cancer control plan led by tribes (like the Cherokee or Choctaw Nations) in collaboration with state public health department could be effective. This might combine all the above: aggressive risk factor modification (through tribal health programs), improved insurance enrollment, mobile screening vans, and patient navigation.

### 8.8. Individual, Community, and Policy Level Interventions

Key interventions discussed in this paper illustrate the necessity of multi-level interventions spanning from individual to community and policy levels for the prevention and management of risk factors, improvements in early detection and in healthcare delivery, and a reduction in the gap in the kidney cancer burden.

#### 8.8.1. Successful Individual-Level Interventions Require Community and Federal Support

Many interventions to reduce modifiable risk factors, such as smoking, obesity, diabetes and hypertension, and screening for kidney cancer require individual behavioral changes. While studies investigate effective and culturally appropriate intervention approaches, individual-level behavioral change is more successful when culturally tailored programs are initiated by local communities and supported by Federal agencies and programs are available at local community organizations, tribal health departments, or IHS clinics. For example, IHS offer various smoking cessation programs. The IHS also administers SDPI-DP supporting programs in over 300 communities.

For the development of new interventions or programs, AI/AN communities and Tribal Nations must be included in each step of the process, from developing programs and interventions, to selection and recruitment of participants, dissemination of findings, protection of data when shared or stored, and respect the principles of Indigenous data governance [174]. These actions are necessary given the mistrust of healthcare systems and research within tribal communities that still lingers from malpractice such as the incident which occurred at Arizona State misusing genetics data from the Havasupai Tribe [173]. Recent program successes in cancer research with Tribal Nations have been emphasizing community-based participatory research that respects the sovereign status of tribes. In one case, Native American Cancer Prevention, a joint research partnership between researchers at Northern Arizona University and University of Arizona worked with Navajo Nation to ensure that their breast and cervical cancer-prevention programs were maintained even during the COVID-19 pandemic [175].

#### 8.8.2. Policy Level Interventions

In addition to allocating Federal funding for tobacco-cessation and diabetes programs, policy-level changes are necessary. EPA funding can be used to remove contaminants, but stricter regulations to reduce environmental exposure and contaminations in tribal lands are necessary. Increased funding for IHS and tribal health facilities, such as funding for Purchase/Referred Care program, which allows those who receive IHS care to get outside medical services when IHS cannot provide, is necessary for improving healthcare access and quality, including having specialized care, improving referral processes, and initiating telehealth and patient navigator programs. However, as recently as 2022, referred-care programs denied or deferred nearly $552 million in expenses for roughly 120,000 requests from eligible patients [176].

Furthermore, policy changes may be necessary for Medicaid, Medicare, and other insurance to cover ultrasound or CT scans for high-risk AI/AN individuals with potential kidney cancer symptoms, expanded low-dose CT scans to lower abdomen during lung cancer screening, and other care necessary for early detection and treatment. As a result of the 2010 Indian Healthcare Improvement Act, IHS-funded facilities can charge Medicaid for services. This has been used as a way to makeup funding deficits, with some IHS service areas having their populations go from 85% uninsured to as low as 10% now after the Medicaid reform. This has been a major success in providing essential care to AI/AN populations and ensuring the continuation and expansion of healthcare service providers [177].

## 9. Conclusions

A combination of prevention, early detection, and healthcare system improvements is needed to close the kidney cancer gap between AI/AN and other populations. Eliminating modifiable risk factors such as tobacco and obesity has proven mitigation strategies but requires sustained commitment and adaptation to Native cultures. Healthcare access improvements can also facilitate earlier detection and treatment initiation, thereby reducing mortality. Importantly, efforts must be community-led or co-led; interventions are most successful when healthcare providers and healthcare policy-makers respect tribal sovereignty and cultural values. For example, involving tribal elders and healers in crafting health messages or integrating Western medicine with traditional practices can enhance trust and uptake [178,179]. Without continued investment, many of these programs may stall or regress—threatening to widen, rather than narrow, the kidney cancer disparity [180]. The failure to fund proven interventions may lead to fewer early diagnoses, more preventable deaths, and a worsening of long-standing disparities in cancer outcomes for AI/AN communities [181]. Finally, measuring progress via data is essential. Continued efforts to improve cancer surveillance in AI/AN populations (like the linkage of IHS data to state registries) will help track whether these interventions are reducing incidence and mortality over time, while the ownership of data at tribal nation levels will help the development of programs for education and outreach, chronic disease prevention and management, and improving healthcare access in AI/AN local communities. Early signs from comprehensive initiatives (e.g., declines in diabetes in some IHS service areas) are encouraging. By addressing both the upstream causes (SSDOH and risk factors) and the downstream factors (diagnosis and treatment), it is possible to significantly reduce kidney cancer incidence and deaths in AI/AN populations in the coming years. Each percentage drop in smoking or each successful early surgery will translate into lives saved and a narrowing of this long-standing health disparity.

## Figures and Tables

**Table 1:** Disparities in modifiable risk in American Indian/Alaska Native (AI/AN) adults.

Risk Factors	Attributable or Relative Risk ^1^	Prevalence
Obesity	30–35% [9,36,37,38]	40.9% of AI/AN vs. 33.4% total population in the U.S. [39]
Tobacco Use	16–20% [37,40]	16–35% in AI/AN vs. 11–13% in White [41]
Hypertension	27% [42,43]	32.9% of AI/AN vs. 27.6% of White in Arizona [44]
Diabetes	40–70% [45,46,47] ^2^	13.6% in AI/AN vs. 10.0 in total population [48]
Chronic Kidney Disease, End Stage Renal Disease (ESRD)	8–10% [42,49]	3346 vs. 1442 ESRD cases per million persons in AI/AN and White [50]

^1^ Attributable and relative risk estimated based on White or general populations, not specifically in AI/AN populations. ^2^ Relative risk.

**Table 2:** Kidney cancer incidence and mortality rates in American Indian/Alaska Native by Indian Health Service Regions.

Region ^1^	Sex	Incidence Rate ^2^	RR ^2^	Mortality Rate ^3^	RR ^3^
Northern Plains	Males	64.8	2.37 *	13.1	2.06 *
	Females	32.3	2.44 *	6.3	2.09 *
Alaska	Males	37.7	1.69 *	12.2	2.17 *
	Females	23.1	1.94 *	7.7	3.36 *
Southern Plains	Males	52.8	2.13 *	13.7	2.01 *
	Females	31.1	2.18 *	5.7	1.75 *
Pacific Coast	Males	29.7	1.39 *	7.2	1.23
	Females	15.4	1.49 *	4.4	1.63 *
East	Males	19.2	0.81	6.5	1.08
	Females	14.2	1.24	4.0	1.49
Southwest	Males	39.9	2.05 *	11.3	2.16 *
	Females	19.5	2.08 *	5.4	2.25 *
U.S. Overall	Males	40.4	1.79 *	10.9	1.83 *
	Females	22.1	1.98 *	5.4	1.99 *

^1^ Purchased/Referred Care Delivery Aras, ^2^ kidney cancer incidence rate (1999–2020) and rate ratio (RR) compared with White from Melkonian et al. [10]. ^3^ Kidney cancer mortality rate (1990–2009) and RR compared to White from Li et al. [20]. * Indicates statistically significantly increased RR compared with White (*p* < 0.05). Incidence and mortality rates are age-adjusted per 100,000 population.

**Table 3:** Key interventions and programs aimed at reducing the incidence and mortality of kidney cancer in the AI/AN population.

Intervention Category	Intervention Examples/Programs	Effectiveness	Feasibility and Implementation Considerations	Recent Efforts and Initiatives
Tobacco Control and Cessation Programs	Culturally tailored smoking cessation programs distinguishing sacred/traditional tobacco from commercial use; counseling; nicotine replacement therapy; community-driven campaigns.	Reduces RCC and other cancer risks; risk declines after quitting (may take >10 years); given high prevalence (up to 1 in 3 adults), even modest reductions yield impact.	Challenges: social norms, addiction; feasibility supported by IHS cessation programs, CDC’s ‘Tips From Former Smokers’ AI/AN testimonials; enforce smoke-free policies; youth engagement.	Great Plains Tribal Cancer Control Program (2023–2027) targeting high smoking rates [137]; Cherokee Nation Cancer Registry [138,139]; ICON initiative in Oklahoma integrating cessation, navigation, tele-oncology [140,141]. Roswell Park Comprehensive Cancer Center Department of Indigenous Cancer Health has multiple lung cancer health prevention programs, including work in workplace health and the tobacco quit line [142].
Obesity and Diabetes Prevention Initiatives	National Diabetes Prevention Program (DPP) adapted for AI/AN; IHS Special Diabetes Program for AI/AN; promote healthy traditional diets and physical activity.	Lifestyle changes reduce weight by 5–7%, cut diabetes incidence, and likely reduce obesity-related cancers.	Needs sustained funding, community buy-in; feasibility via tribal health departments, community health workers, cultural integration (traditional dance, gardens); address food insecurity.	Special Diabetes Program for Indians reduced ESRD due to diabetes by 54% (1999–2013); diabetes prevalence declined 2013–2017 [143].
Hypertension and Kidney Disease Management	Blood pressure screening at every visit; aggressive blood pressure management; culturally acceptable patient education; early CKD treatment; ultrasound screening for CKD/dialysis patients.	Good blood pressure control and ACE inhibitors protect kidneys, may lower RCC risk indirectly.	Requires improved access and IHS/tribal clinic resources; task-shifting to community health reps; telemedicine consults.	Target: BP™ initiative supporting Federally Qualified Health Centers including AI/AN-serving sites; part of National Hypertension Control Initiative (HRSA & Office of Minority Health grant) training 350 health centers; 3100+ organizations served 8 million patients (42% ≥70% control) [144].
Environmental Health Interventions	Arsenic-free water provision; under-sink filtration; remediation of uranium mine waste; occupational safety for mining industries.	Eliminates carcinogen exposure, reducing kidney cancer and other disease risks (arsenic increases RCC risk by 6–22%).	Infrastructure projects feasible with government/tribal support; workplace safety via OSHA enforcement and tribal advocacy.	EPA Ten-Year Plan (2020–2029) for uranium contamination cleanup on Navajo Nation [81]; Strong Heart Study partnerships [80,145].
Improving Healthcare Access and Quality	Increase IHS/tribal facility funding; bring specialists via outreach/telehealth; expand Medicaid; patient navigation; culturally competent outreach.	Better resources enable earlier diagnosis/treatment; insurance improves survival via earlier access.	Policy changes, partnerships; navigation programs hire from within communities; respect traditional healing.	Medicaid expansion in some AI/AN states [146]; tribal-state Medicaid collaborations [147]; University of Oklahoma Stephenson Cancer Center tribal navigation project [148]; Roswell Park Indigenous & Rural Patient Navigation Program [149]; CANOE Partnership [150]; Messengers for Health (Crow Reservation) [151].
Early Detection Strategies	Incidental detection via imaging; targeted screening (e.g., ultrasound in high-incidence AI/AN regions); strengthen other cancer screenings.	Potentially detects RCC earlier in high-risk groups; cost-effectiveness unproven in general population.	Requires planning, resources, community acceptance; ultrasound is non-invasive and acceptable.	Cherokee Nation Cancer Registry [138,139]; CDC–IHS data linkage improving AI/AN cancer reporting accuracy [10].
Interventions in High-Risk Communities	Localized interventions for specific areas (e.g., food/nutrition programs, tele-oncology, environmental monitoring, smoking reduction policies).	Tailored to address unique local risk factors; integrates multiple strategies for higher impact.	Needs collaboration between tribes, local health systems, and public health agencies.	San Manuel Indian Health Clinic programs in Southern California [152]; Northern Plains environmental/tele-oncology efforts [82,153,154]; Oklahoma tribal cancer control plans [3].

## Data Availability

Kidney cancer incidence rates to show incidence trends were obtained from North American Association of Central Cancer Registries (NAACCR) Cancer in North America (CiNA) Explorer (https://www.naaccr.org/interactive-data-on-line/, accessed on 25 February 2026). The prevalence of major modifiable risk factors for kidney cancer in AI/AN adults were obtained from National Health Interview Survey (NHIS) and Kidney Early Evaluation Program (KEEP).

## Abbreviations

The following abbreviations are used in this manuscript:

ACS	American Cancer Society
AI/AN	American Indian and Alaska Native
AUC	Area under the curve
CANOE	Cancer Awareness, Navigation, Outreach, and Equitable Indigenous Health
CDC	Centers for Disease Control and Prevention
CiNA	Cancer in North America
CKD	Chronic kidney disease
DPP	Diabetes Prevention Program
EPA	Environmental Protection
ESRD	End-stage renal disease
GLP1	Glucagon-like peptide-1 receptor agonist
HRSA	Health Resources and Services Administration
IHS	Indian Health Service
ICON	Improving Cancer Outcomes in Native American Communities
KEEP	Kidney Early Evaluation Program
NAACCR	North American Association of Central Cancer Registries
NHIS	National Health Interview Survey
NIH	National Institutes of Health
OSHA	Occupational Safety and Health Administration
PRCDA	Purchased/Referred Care Delivery Areas
RCC	Renal cell carcinoma
RMC	Renal medullary carcinoma
RSBCIHI	Riverside–San Bernardino County Indian Health, Inc.
SDPI-DP	Special Diabetes Program for Indians Diabetes Prevention
SEER	Surveillance, Epidemiology, and End Results
SSDOH	Structural and social determinants (or drivers) of health
